# Acoustic combinatorial crystallization generates huge 9216 crystallization library from 64 building blocks

**DOI:** 10.1063/4.0001108

**Published:** 2025-10-27

**Authors:** Alexei Soares

**Affiliations:** Brookhaven National Laboratory, Upton, NY, USA

## Abstract

We present a novel method for protein crystallization: acoustic combinatorial crystallization. In this approach, crystallization cocktails are assembled directly on crystallization labware using acoustic liquid handling, without relying on a pre-defined crystallization libraries. Instead, three distinct building block libraries are acoustically combined combined in all possible permutations (24 precipitants, 24 buffers, and 16 additives). This generates a Sample Preparation On Target (SPOT) library of 9,216 unique crystallization conditions, each assembled in situ in the presence of the protein. This highly diverse, on-demand library increases the likelihood of identifying successful crystallization conditions and enhances lessons learned when crystals are not formed, offering insights into protein stability, solubility, and sensitivity to ionic strength. We demonstrate the advantages of this method through case studies where crystals were obtained using SPOT libraries after conventional crystallization screens were unsuccessful.

